# RNAs: dynamic and mutable

**DOI:** 10.1186/s13059-017-1361-5

**Published:** 2017-11-29

**Authors:** Mihaela Zavolan, Brenton R. Graveley

**Affiliations:** 1 Computational and Systems Biology, Biozentrum, University of Basel, CH-4056 Basel, Switzerland; 2 Department of Genetics and Genome Sciences, Institute for Systems Genomics, UConn Health, Farmington, CT 06030 USA

Why a Special Issue on RNA and Gene Regulation? Until the late 1990s, it was thought that only a small number of ribonucleic acids were stably expressed in every cell: ribosomal RNAs, transfer RNAs, small nuclear RNAs, and small nucleolar RNAs. In contrast, the messenger RNA (mRNA) population was known to be more dynamic and would change in response to signals and cell state. Around two decades ago, a much more diverse RNA world began to emerge, populated with many different types of RNAs, from very short to very long, very transient to very stable, associating with different proteins and carrying out many new functions. This Special Issue of *Genome Biology* is on a specific, though still very broad aspect of this diverse landscape: RNA modification and its role in gene regulation.

The discovery of mRNA involved a long and fascinating struggle by many scientists [1]. Evidence that regulatory RNAs exist became apparent from the use of many experimental systems [2]. Similarly, the multitude of modifications that have been found on naturally occurring ribonucleotides has been known for many years [3]. What then makes these research fields so active today? One of the main reasons for the resurgence in these fields is the breakthrough in technologies to probe cellular content on a global scale. Despite the still large imprecision of individual measurements, patterns can be readily distinguished. A second important factor is the development of ingenious approaches to enrich specific RNA populations. Many of the articles in this Special Issue illustrate these points.

He and colleagues [4] review the far-reaching implications of RNA modification for cellular function. One of the most common modifications is the deamination of adenosine to inosine (A-to-I) in double-stranded RNA (dsRNA). This modification renders RNAs unstable and alters their base-pairing properties, which has implications for RNA processing and decoding into proteins, reviewed here by Walkley and Li [5]. The adenosine deaminating enzymes presumably evolved early in the metazoan lineage, and their functions have been surveyed by Levanon and colleagues [6]. Park et al. [7] describe that, as expected, there is much variation in A-to-I editing between human individuals, but as yet the consequences of this editing have been poorly characterized. Nevertheless, modulatory elements have been identified and some of these are described by Daniel et al. [8]. However, the article by Heraud-Farlow et al. [9] disputes the importance of A-to-I-mediated changes in protein sequences for organism function, and emphasizes that the impact of this modification remains to be fully understood.

The complexity of interactions and functions from relatively simple sequence elements continues to unfold. The study by Patel et al. [10] highlights a specific genetic mutation that generates an AU-rich element with pathogenic consequences. Understanding the tissue-dependent dynamics of individual mRNAs remains a challenge, as many RNA binding proteins (RBPs) modulate these dynamics in unexpected ways. Rissland et al. [11] bring an additional piece to this puzzle, and describe how microRNAs (miRNAs) modulate the interactions of RBPs with target mRNAs. How translation initiation changes in response to stress is discussed by Costello et al. [12] who studied eIF4F-mRNA interactions. Meyer et al. [13] used crosslinking and immunoprecipitation to map the binding sites of the plant clock-regulated AtGRP7 protein.

Non-coding RNAs are a heterogeneous class of RNAs in terms of transcription requirements, sequence length, and localization, and their functions are correspondingly diverse. An up-to-date review of the mechanisms of non-coding RNAs is presented by Marchese, Raimondi, and Huarte [14]. One of the earliest described functions of long non-coding RNAs (lncRNAs) was in the regulation of chromatin structure [15]. In this Special Issue, Marín-Béjar et al. [16] identified a conserved sequence element that mediates the interaction with polycomb repressor complex 2 (PRC2), through which the LINC-PINT lncRNA (long intergenic non-protein coding RNA, p53 induced transcript) regulates tumor invasion. A large number of non-coding, antisense transcripts have been identified following the infection with Herpes simplex virus 1; induction of antisense transcription provides protection against apoptosis as described by Wyler et al. [17].

Finally, two articles in this Special Issue contribute to the current debate on the functions of alternatively spliced transcript isoforms. Echoing earlier studies that indicated a high degree of stochasticity in splice site choice [18], Saudemont et al. [19] argue that observed splicing patterns reflect the fitness cost of mis-splicing. Finally, Schmitz et al. [20] evaluate the prevalence of intron retention in vertebrates and suggest that this mechanism enhances regulatory complexity, though the evidence for this remains very limited.

More articles on these topics will be added in the coming weeks. Collectively, the articles contained within this Special Issue of *Genome Biology* touch on and provide new insight into many of the key issues in the broad and exciting field of RNA regulation.
